# Implementation of a Decision Support Algorithm for Multidisciplinary Teams: Initial Case Comparisons

**DOI:** 10.1093/geroni/igaf122.225

**Published:** 2025-12-31

**Authors:** Zachary Gassoumis, Erin Thayer

**Affiliations:** UTHealth Houston, Houston, Texas, United States; University of Southern California, Los Angeles, California, United States

## Abstract

Adult maltreatment multidisciplinary teams (MDTs) bring together professionals from health, social service, and justice systems to address complex cases of abuse, neglect, and self-neglect of older adults and younger adults with disability. MDTs are a promising intervention with demonstrated system outcomes, but they experience challenges identifying cases for review. In partnership with San Francisco Adult Protective Services (APS), we developed and implemented an algorithm to identify APS cases that appear appropriate for MDT review. Once identified by the algorithm as being potentially appropriate for an MDT, cases were randomized to a control group or for recommendation to APS staff as a decision support resource. This study attempts to better understand the algorithm-selected cases by comparing the control group with all cases presented to the MDT from October 2024 onward, using Fisher’s Exact and Wilcoxon Rank Sum tests. Preliminary results (N = 58) include that MDT and control cases had comparable reduction in risk from case opening to closure (p=.70), but MDT cases were significantly more likely to be closed due to receiving services from other agencies (32% vs. 10%, p=.048). MDT cases were somewhat, though not significantly, less likely to have clients who refuse all interventions (0% vs. 15%, p=.063). These results provide insight into the operation of MDTs in the presence of this decision support tool, suggesting potential targets for further analyses. This research is one piece in a move toward developing and testing practice enhancement for APS and MDTs, as a way to improve outcomes for elder mistreatment cases.

